# Colors and Handles: How Action Primes Perception

**DOI:** 10.3389/fnhum.2021.628001

**Published:** 2021-05-11

**Authors:** Marcello Costantini, Davide Quarona, Corrado Sinigaglia

**Affiliations:** ^1^The Embodied Adaptive Mind Laboratory (TEAM Lab), Department of Psychological, Health and Territorial Sciences, University G. d’Annunzio, Chieti, Italy; ^2^Institute for Advanced Biomedical Technologies—ITAB, University G. d’Annunzio, Chieti, Italy; ^3^Department of Philosophy, Faculty of Humanities, University of Milan, Milan, Italy; ^4^Cognition in Action Unit, PhiLab, University of Milan, Milan, Italy

**Keywords:** action and perception, color discrimination, motor priming, affordances, two visual systems

## Abstract

How deeply does action influence perception? Does action performance affect the perception of object features directly related to action only? Or does it concern also object features such as colors, which are not held to directly afford action? The present study aimed at answering these questions. We asked participants to repeatedly grasp a handled mug hidden from their view before judging whether a visually presented mug was blue rather than cyan. The motor training impacted on their perceptual judgments, by speeding participants’ responses, when the handle of the presented mug was spatially aligned with the trained hand. The priming effect did not occur when participants were trained to merely touch the mug with their hand closed in a fist. This indicates that action performance may shape the perceptual judgment on object features, even when these features are colors and do not afford any action. How we act on surrounding objects is therefore not without consequence for how we experience them.

## Introduction

Action and vision are linked. There is much evidence of visual perception priming action. To illustrate, the sight of a teapot or a handled mug has been shown to make people faster when performing a compatible action (Ellis and Tucker, 2000; Tucker and Ellis, 2001, 2004; Costantini et al., 2010). Conversely, several studies indicated that action may affect visual perception. For instance, action preparation has been found to facilitate the visual detection of an object, when its shape and orientation are congruent with respect to the prepared action (Craighero et al., 1999). Interestingly, this facilitation effect has been demonstrated to occur not only at short-time scales but also at long-time scales, with motor learning enhancing perceptual judgment on congruent movement patterns (Hecht et al., 2001). Finally, there is evidence that this effect is primarily due to the impact of action on how congruent objects are perceived, rather than merely the induction of a response bias (Cardoso-Leite et al., 2010).

A consensus account points to common coding for action and perception: planned movements are represented in the same format as the distal perceptual effects they evoke in the environment (Prinz, 1990; Hommel et al., 2001). This would explain both why the visual perception of objects with a given shape and orientation can afford congruent actions, even in absence of any intention to act, and why acting upon these objects can affect the perceptual judgments about their shape and orientation.

The notion that object features may directly afford a variety of actions to individuals who are able to act upon them dates back to Gibson (1979). Post-Gibsonian attempts to further characterize this notion related to object affording features such as size and shape to motor abilities (Chemero, 2003; Costantini and Sinigaglia, 2012), distinguishing between micro- and macro-affordances (Ellis and Tucker, 2000) as well as between “canonical” and “affordance in general,” with the former incorporating the established, widely agreed upon function of objects (Costall, 2012).

Neurophysiological and neuroimaging studies have provided these notions with a neuronal counterpart, showing that micro-affordances and canonical affordances can be encoded by premotor (area F5) and parietal (area AIP) neurons. These neurons selectively respond not only during the execution of specific hand and mouth actions, but also during the observation of object features such as size and shape functionally congruent to those actions (Murata et al., 1997, 2000; Chao and Martin, 2000; Grèzes et al., 2003). These neurons, named *canonical neurons*, have been claimed to transform the visual information concerning critical object features such as shape and orientation into the motor representation of the appropriate action movements (Jeannerod, 1995).

While the common coding for action and perception has been extensively investigated, less is known about whether such coding only pertains to the object features directly related to action rather than extending to object features held to not directly afford action. A paradigmatic example of the latter object features are colors. They can be relevant for action, of course. If you would like to taste a tea with your favorite red mug, color discrimination will be critical for the identification of the mug and therefore for the success of your action. Nevertheless, colors have been shown to not directly afford action as other object features such as shape and orientation do (Tipper et al., 2006).

Although colors do not seem to directly afford any specific action, there is evidence that color perception might somehow affect action. For instance, color perception has been found to enhance the force and the velocity of a hand grip (Green et al., 1982; Keller and Vautin, 1998; Elliot and Aarts, 2011). Even more interestingly, Gentilucci et al. (2001) demonstrated that colors might differentially affect the kinematic components of reaching and grasping actions. Indeed, while the color of some stimulus distractors selectively influenced reach components such as arm peak acceleration, maximal deviation of arm trajectory, and reaching time, the color of the target-object critically impacted on a crucial grasp component such as maximal grip aperture.

These findings are in line with the evidence of cross-talk between the dorsal and the ventral visual stream areas involved in hand action control and execution (van Polanen and Davare, 2015; Milner, 2017). Notably, it has been shown that parieto-frontal areas have rapid access to object feature information stored in the ventral stream areas which is critical to identify a target object (Borra et al., 2008). Interestingly, AIP neurons have been shown to receive color information from area V4 *via* the medial superior temporal cortex and their responses have been demonstrated to be modulated by the color of an action-related cue (Baumann et al., 2009).

Taken together, these findings suggest that the dorsal and the ventral visual stream areas may share a representation integrating motor and perceptual object features (Perry and Fallah, 2014), with such an integration explaining why color perception might affect action performance. This naturally raises the question of whether the converse is true as well. Does action performance somehow affect color perception? Can the integrated representation of object features be effective not only when acting upon an object but also when perceptually judging its color? These questions remained largely unanswered. Filling this gap was the main aim of the present study.

We adapted a visual detection paradigm previously used to investigate whether and how action performance may influence the perception of an object affording feature such the presence of a handle on a mug (Costantini et al., 2019). In our main experiment (Experiment 1), participants were given motor training consisting in repeatedly reaching for, and grasping with their right hand, a handled mug hidden from their view. Immediately after, they were asked to judge the color of a visually presented mug (e.g., blue or cyan). The visually presented mug could be with or without a handle. In the former case, the handle could be right- or left-oriented. In a control experiment (Experiment 2), participants undertook the perceptual judgment task after motor training consisting in reaching for and merely touching the body of the mug with their right hand closed in a fist.

The training should provide participants with a representation of the affording features of the mug which were critical for the successful performance of the required actions. In the case of the reach-to-grasp training, the represented feature was its right-oriented handle (which was needed for preparing and executing a suitable grip). In the case of the reach-to-touch training, participants needed to represent the location and the resistance of the mug, but neither its shape nor the orientation of its handle. Our conjecture was that these object representations might integrate motor and perceptual features. If this conjecture is correct, the object representations evoked during motor training should also be effective during the perceptual task, affecting participants’ performance in color detection. And this is what we actually found. Indeed, after repeatedly reaching for the handle of a mug and grasping it with their right hand, participants were faster in perceptually judging the color of the visually presented mug when its handle was right-oriented (and thus aligned with the hand used in the reach-to-grasp training) than when it was either left-oriented or absent. This compatibility effect did not occur in the perceptual task after the reach-to-touch training.

## Materials and Methods

### Participants

Fifty participants (seven males, mean age 21.2) were enrolled and randomly distributed between the two experiments. All of participants had normal or corrected-to-normal vision, were right-handed as self-reported, and were naïve as to the purposes of the experiments and gave their informed consent. Informed consent was signed by all the participants before starting the experiment. The study was approved by the local ethics committee and was conducted in accordance with the ethical standards of the 1964 Declaration of Helsinki.

## Experiment 1

### Stimuli and Procedure

**Figure 1:**
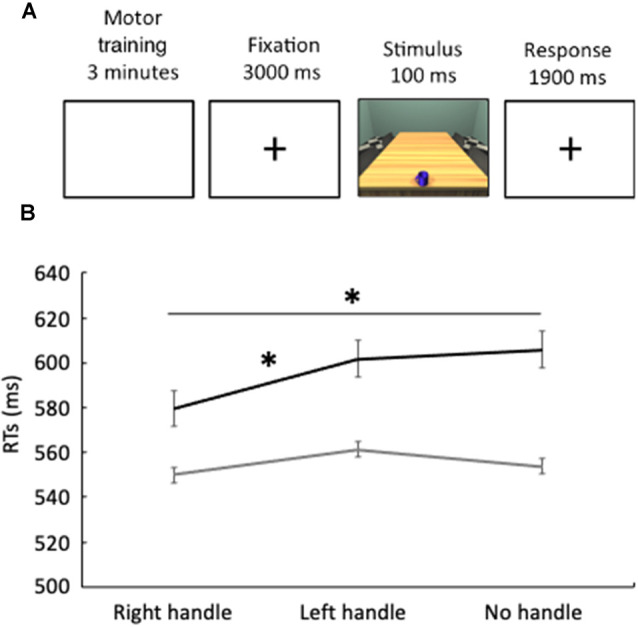
**(A)** Temporal structure of Experiments 1 and 2. **(B)** The black line represents reaction times, expressed in milliseconds, in Experiment 1 (reach-to-grasp), while the gray line represents reaction times in Experiment 2 (reach-to-touch). Error bars indicate standard errors. *p < 0.05.

In Experiment 1 visual stimuli consisted of six images (1,024 × 768 pixels) depicting a 3D room with a table and a mug on it, created by 3DStudioMax v.13 (Figure 1A). The mug could be either handled or without a handle and had two different colors: blue and cyan. Therefore, there were three blue mugs (a right-oriented handled mug, a left-oriented handled mug, and a mug without a handle) and three cyan mugs (a right-oriented handled mug, a left-oriented handled mug, and a mug without a handle).

The images were presented with Matlab software. Trials were divided into four balanced blocks of 45 images randomly presented, therefore, there were 180 trials in total (45 trials × 4 blocks). Subjects sat comfortably on a chair in front of a computer screen. The experiment consisted in motor training immediately followed by a perceptual task.

In the motor training, participants were asked to repeatedly reach for and grasp the handle of a mug, positioned 25 cm from their body midline, with their right hands. The mug’s handle was oriented toward the right. Participants were instructed to grab the mug by the handle and lift it. The grasping hand always started from the same instructed position, with the thumb and index forming a pinch grip. After each reach-to-grasp movement participants had to return to the starting position.

The motor training lasted for 3 min. Each reach-to-grasp action took approximately 1,500 ms. This means that participants performed on average 120 reach-to-grasp actions. A black box covered both the participant’s arm and the mug during the motor training, thus preventing them from visually accessing their reach-to-grasp actions. The correct execution of the motor training was monitored by an experimenter seated on the opposite side. As far as the perceptual task is concerned, all the 180 stimuli were presented for 15 min. The stimulus was preceded by a blank screen with a fixation cross lasting 3 s. The image was presented for 100 ms followed by a blank screen with a fixation cross lasting 1,900 ms, during which responses were collected (Figure 1A). Participants were asked to judge the color of the mug, by saying “yes” for the blue mug and “no” for the cyan mug as soon as the mug was presented. Accuracy and vocal latency times of each single response were recorded. Vocal latency times were computed from the initial presentation of stimulus to the start of verbal utterance.

### Results

Accuracy was higher than 98% in all the conditions, thus not further analyzed. Erroneous responses were not included in the analyses. Shapiro–Wilk’s tests (*p* > 0.05) and visual inspection of the data showed that reaction times were not normally distributed. Indeed, they showed a skewness of 1.80 (*SE* = 0.47) and a kurtosis of 4.45 (*SE* = 0.80). Wilcoxon tests revealed faster reaction times (*z* = 2.38; *p* = 0.017, Figure 1B, Table 1) in the perceptual judgment task when the handle of the mug was oriented to the right (*RT* = 580 ms ± 106) than when it was oriented to the left (601 ms ± 100). Furthermore, reaction times were faster (*z* = 2.41; *p* = 0.016, Figure 1B, Table 1) when the handle of the mug was oriented to the right (*RT* = 580 ms ± 106) than when the mug had no handle (605 ms ± 104). Such differences indicate a priming effect.

**Table 1:** All the statistical comparisons of interest.

	Z	*p*-level
**Reach-to-grasp**		
Right handle—Left handle	2.38	0.017
Right handle—No handle	2.41	0.016
Left handle—No handle	0.28	0.77
**Reach-to-touch**		
Right handle—Left handle	1.14	0.25
Right handle—No handle	0.60	0.55
Left handle—No handle	1.25	0.26

## Experiment 2

### Visual Stimuli and Procedure

Differently from Experiment 1, participants were instructed to repeatedly reach for and merely touch the body of the mug with their right hand closed in a fist. The remaining setting of the motor training and perceptual task were as in Experiment 1.

### Results

Shapiro–Wilk’s tests (*p* > 0.05) and visual inspection of the data showed that reaction times were not normally distributed. Indeed, they showed a skewness of 1.60 (*SE* = 0.51) and a kurtosis of 4.60 (*SE* = 0.90). Wilcoxon test revealed no significant differences between experimental conditions (all *p*s > 0.25, Figure 1B, Table 1).

## Discussion

The aim of the present study was to explore how deeply action may influence perception. More specifically, we assessed the influence of action performance on the perceptual judgment of object features which do not directly afford action, such as color. To this purpose, we asked participants to judge whether a visually presented mug had a blue or cyan color just after motor training consisting in repeatedly reaching for and grasping with their right hand a handled mug hidden from their view (Experiment 1). The same perceptual judgment task was undertaken just after motor training in which participants had to reach for and merely touch the mug (Experiment 2).

The main finding concerns the priming effect induced by the repeated performance of a reach-to-grasp action on perceptual judgment about color. Indeed, participants were significantly faster in judging the color of right-oriented handled mugs than that of both left-oriented handled and non-handled mugs. Although there was no significant interaction between the main experiment (Experiment 1) and the control experiment (Experiment 2), it is worth noting that the priming effect was not reported when the perceptual judgment task was undertaken just after the reach-to-touch motor training.

This finding is in line with the notion of common coding between action and perception (Prinz, 1990; Hommel et al., 2001). According to this notion, action and perception would share a common representation format. Perceiving an action effect would recruit the same representation as performing the associated action; conversely, performing an action would involve the same representation as perceiving the associated effect. Now, there is evidence that actions are facilitated by the perception of object features associated with them (Ellis and Tucker, 2000; Tucker and Ellis, 2001, 2004) as well as that perceptual judgments about object features are primed by previous actions (Craighero et al., 1999; Hecht et al., 2001; Cardoso-Leite et al., 2010). Our finding extends this evidence to a perceptual feature, such as color, that goes beyond the association between the action and its perceptual effects. Indeed, color was not an action effect targeted by the motor training, in which participants repeatedly reached for and grasped (or touched) the mug without seeing it (and without seeing their grasping or touching hand).

In demonstrating that action may affect color perception, our study complements previous studies suggesting that color perception might affect action performance. In particular, it has been shown that color perception might modulate how people shape their hand in order to reach for and grasp a target object (Gentilucci et al., 2001). Our results indicate that this modulation may also occur in the opposite direction, with grasping action affecting how people perceptually judged the color of a viewed graspable object.

These results might seem at odds with Tipper et al.’s (2006) findings. Indeed, they compared the priming effects in two different discrimination tasks concerning either the shape (e.g., square or round) or color (e.g., blue or green) of an object such as a door handle. The results showed that participants were faster in discriminating the shape of the handle when the latter was right-oriented than when it was left-oriented. Interestingly, participants were even faster when they had to discriminate the shape of a right-oriented depressed handle where the depression clearly suggested someone had turned it. Neither effect was found when participants had to discriminate the color of the handle.

However, the conflict between our finding and Tipper et al.’s (2006) results is very apparent. While Tipper et al. (2006) focused on the action-state of the target object, we decided to manipulate the action-state of the participants. In contrast to Tipper et al. (2006), in our study participants performed the color discrimination task just after being motorically trained to reach for and grasp an object. Consistently with the common coding principle, a likely hypothesis is that the motor training provided participants with a representation of the affording features of the targeted object (e.g., the presence of a right-oriented handle), which could be used for the perceptual task, thus enhancing participants’ performance in judging colors when the visually presented mug was aligned with its handle to the trained hand. This could also explain why this effect did not occur after reach-to-touch motor training. This training did not require the representation of the affording features (e.g., the presence of a handle and its possible orientation), which were manipulated in the perceptual task by the different visual presentations of the object (a mug with a right- or left-oriented handle or without any handle).

The notion of an object representation that would integrate motor and perceptual features is consistent with the evidence displaying mutual cross-talk between the dorsal and the ventral visual streams (Milner, 2017). For instance, it has been reported that patient D.F., who suffers from a visual form agnosia and exhibits severe difficulties in discriminating object shapes and orientations, performed the discrimination tasks better than would have been expected when she could somehow act upon the target object (Schenk and Milner, 2006). Notably, she significantly improved in recognizing the shape of an object while grasping it, with this effect being mainly due to the sensorimotor transformations involved in the grasping action rather than mere proprioceptive and reafferent cues.

These and other similar behavioral data have been provided with an anatomical counterpart by a large number of studies demonstrating direct connections between dorsal and ventral stream areas, which are particularly relevant for monitoring and controlling grasping action. While the dorsal stream would retrieve object identity information stored in ventral stream areas, the ventral stream might process action-related information from dorsal stream areas to refine its object representation. This view has been supported by an fMRI monkey study showing that the inactivation of sulcal territories of the posterior parietal cortex determined a reduced activity both in the parietal and the inferotemporal cortex implicated in 3D object information processing, thus provoking a perceptual deficit in a depth structure categorization task (Van Dromme et al., 2015).

Interestingly, there is evidence that the object representation shared by the dorsal and the ventral areas might also concern an object feature such as color (Perry and Fallah, 2014). Indeed, the medial superior temporal cortex has been shown to receive color information from visual area V4 and send it to the anterior intraparietal area (AIP), which is a critical node of the parieto-frontal network, containing purely visual and canonical neurons activated by specific object-directed hand actions such as grasping and manipulating (Murata et al., 2000; Borra et al., 2008; Lanzilotto et al., 2019). It is worth noting that AIP has been shown to contain neurons encoding specific hand grasping movements directed to perceived objects on the basis of the color of the presented cue (Baumann et al., 2009). The same AIP neurons responded to the color of the cue also in the absence of the grasp target, in line with the our hypothesis of an integrated object representation of motor and perceptual features, shared by the dorsal and the ventral stream areas.

This hypothesis could also explain why the effect of action performance on perceptual judgment reported in the present study was the opposite of that we found in a previous study (Costantini et al., 2019). In that study we took advantage of a motor-sensory adaptation paradigm. According to this paradigm, the repeated performance of an action would induce an adaptation in the premotor and motor brain areas, which should result in a loss of function of visual perception of action-related features congruent with the motor training (Cattaneo et al., 2011). Participants were asked to judge whether a visually presented mug was handled or not immediately after motor training consisting in repeatedly grasping the handle of a mug hidden from their view. The results showed that they were slower in making a perceptual judgment when the handle of the visually presented mug was aligned with the trained grasping hand. Our conjecture was that this effect was likely due to the adaptation of the canonical neurons. Because these neurons are triggered from both motor and visual congruent inputs (Murata et al., 1997, 2000), the effects of their firing history driven by motor execution could be observed in the visual domain, with a slowing-down of performance when the perceptually judged object features were the same as in the motor training.

Unlike the previous study, here the perceptual judgment concerned an object feature, the color of the visually presented mug, that was not targeted by motor training. This is the reason why one should not have expected any motor-sensory adaptation. On the contrary, the motor representation of the right-oriented handle of the mug evoked by motor training could be used for the perceptual judgment, enhancing the detection of the color when associated with a visually presented mug with its handle oriented in same direction as in the motor training. This effect would not be due to the canonical neurons *per se*, but to the mutual transfer of information between the canonical and visual neurons of AIP and the ventral visual stream areas, including area V4, typically involved in encoding color.

Acting upon an object may affect the perceptual judgment of its features, even when these features are not affording any specific actions. This indicates that action and perception may share object representations which can be effective not only in guiding a grasping hand toward a handled mug but also in shaping the perceptual judgment of its color, thus facilitating the discrimination of it. How people act on surrounding objects is not without consequence for how they experience them.

## Data Availability Statement

The raw data supporting the conclusions of this article will be made available by the authors, without undue reservation.

## Ethics Statement

The studies involving human participants were reviewed and approved by University of Milan, Ethics Committee. The patients/participants provided their written informed consent to participate in this study.

## Author Contributions

CS and MC conceptualized and designed research. MC and DQ performed the research and analyzed the data. CS and MC wrote the article. All authors contributed to the article and approved the submitted version.

## Conflict of Interest

The authors declare that the research was conducted in the absence of any commercial or financial relationships that could be construed as a potential conflict of interest.
